# Combined Transcriptome and Lipidomic Analyses of Lipid Biosynthesis in *Macadamia ternifolia* Nuts

**DOI:** 10.3390/life11121431

**Published:** 2021-12-18

**Authors:** Rui Shi, Haidong Bai, Biao Li, Can Liu, Zhiping Ying, Zhi Xiong, Wenlin Wang

**Affiliations:** 1 Key Laboratory for Forest Resources Conservation and Utilization in the Southwest Mountains of China, Ministry of Education, Southwest Landscape Architecture Engineering Research Center of National Forestry and Grassland Administration, Southwest Forestry University, Kunming 650224, China; shirui@swfu.edu.cn (R.S.); liucanswfu@163.com (C.L.); ying15336026063@outlook.com (Z.Y.); 2 Lincang Academy of Forestry, Lincang 677009, China; lcjg2016@163.com; 3 Yuxi Sannong Plateau Characteristic Modern Agriculture Co., Ltd., Chengjiang 652599, China; biaobiao201@163.com; 4 Guangxi South Subtropical Agricultural Science Research Institute, Longzhou 532415, China

**Keywords:** *Macadamia ternifolia*, transcriptome, lipids, fatty acids, genes

## Abstract

Macadamia nuts are considered a high-quality oil crop worldwide. To date, the lipid diversity and the genetic factors that mediate storage lipid biosynthesis in *Macadamia ternifolia* are poorly known. Here, we performed a comprehensive transcriptomic and lipidomic data analysis to understand the mechanism of lipid biosynthesis by using young, medium-aged, and mature fruit kernels. Our lipidomic analysis showed that the *M. ternifolia* kernel was a rich source of unsaturated fatty acids. Moreover, different species of triacylglycerols, diacylglycerol, ceramides, phosphatidylethanolamine, and phosphatidic acid had altered accumulations during the developmental stages. The transcriptome analysis revealed a large percentage of differently expressed genes during the different stages of macadamia growth. Most of the genes with significant differential expression performed functional activity of oxidoreductase and were enriched in the secondary metabolite pathway. The integration of lipidomic and transcriptomic data allowed for the identification of glycerol-3-phosphate acyltransferase, diacylglycerol kinase, phosphatidylinositols, nonspecific phospholipase C, pyruvate kinase 2, 3-ketoacyl-acyl carrier protein reductase, and linoleate 9S-lipoxygenase as putative candidate genes involved in lipid biosynthesis, storage, and oil quality. Our study found comprehensive datasets of lipidomic and transcriptomic changes in the developing kernel of *M*. *ternifolia*. In addition, the identification of candidate genes provides essential prerequisites to understand the molecular mechanism of lipid biosynthesis in the kernel of *M*. *ternifolia*.

## 1. Introduction

Macadamia is a nut tree with high-quality kernel oil. It belongs to the family Proteaceae, known as Australian or sometimes Hawaiian nuts, and has been grown worldwide in tropical and subtropical regions [1,2]. The genus Macadamia has four types of species that are commercially grown to produce nuts. These species are *Macadamia integrifolia*, *M. tetraphylla*, *M. ternifolia*, and *M. jansenii* [3]. However, *M. integrifolia* and *M. tetraphylla* have more significance due to their high-quality oils [4]. Approximately 44,000 metric tons of macadamia kernels are produced per year in the world and 14,100 metric tons per year are produced alone in Australia [5]. Because of its simple production technology, survival in low-fertility soil, high profitability, and low-temperature resistance compared to other traditional crops of tropical regions [6], the planting area of macadamia promptly increased over the past few years worldwide. China is a world-leading country in terms of planting area and is expected to reach more than 15,000 ha in the southern regions. About 90% of China’s total production comes from just Yunnan and Guangxi province [7]. The research knowledge related to breeding tools, nutrition management, disease management, processing methods, and product development is fundamental for the rapid devolvement of the macadamia oil industry around the world [8].

The macadamia is an evergreen nut tree and produces fruits 5–6 years after planting [9]; the fruits are commonly called nuts, and consist of a husk, shell, and kernel [10]. The kernel is a quality product, the edible part, and contains more than 60% lipids [5]. The kernel is consumed fresh, fried, roasted, or caramelized [11]. Interestingly, macadamia nuts are comprised of a high amount of unsaturated fats (70 to 80%) as compared with other edible nuts, specifically oleic and palmitoleic acids [12,13]. The high rate of palmitoleic acids can be utilized as biofuel to reduce issues of energy availability [14]. However, fuel properties, such as the cetane number, kinematic viscosity, oxidative stability, cold flow, and lubricity, in *M. ternifolia* still need exploration. The high percentage of unsaturated fats maintained lower blood cholesterol and ultimately prevent chronic diseases, e.g., cardiovascular diseases, cancer, and dyslipidemia in humans [15,16,17]. Because of these health benefits, macadamia nuts, together with walnuts, peanuts, and almonds, are named as heart-healthy foods by the Food and Drug Administration [18]. Moreover, macadamia nuts are a vital source of minerals (calcium, iron, phosphorus, magnesium, and potassium) [4], vitamins (thiamine, riboflavin, retinol, and niacin), carbohydrates, protein, and fiber [13]. However, the nut nutrient characteristics generally depend on the species, cultivar, growing conditions, location, and management practices [19]. The refined oil obtained from macadamia nut is pale yellow and is widely utilized in the cake and cookies industry [20]. Besides this, macadamia oil is used in the pharmaceutical industry but also has potential applications in the cosmetics industry to produce skin-cleaning and -lightening agents [21].

In terms of various interests, plant lipids are the primary source of energy and essential fatty acids and they have useful industry applications and a significant impact on the economy and human diet around the world [22]. Natural fatty acids are divided into saturated or unsaturated forms. Most saturated fatty acids have a hydrocarbon chain with a single bond, high melting point, and are solid at room temperature. In contrast, unsaturated fatty acids have a hydrocarbon chain with multiple bonds, low melting points, and are liquid at room temperature [23]. In recent years, high-throughput sequencing has become a routine task to identify putative candidate genes for a specific trait or character [24,25]. The lipids profile is a major determinant of oil quality during storage and processing. In this regard, high-quality RNA-seq research was utilized to understand the genetic mechanisms of lipid regulation in Arabidopsis [26], soybean [27], *Brassica napus* [28], jatropha [29], peanut [30], peach [31], castor bean [32], and pecan [33]. Research in oilseed crops identified that genes associated with 3-ketoacyl-acyl carrier protein synthase (KAS), beta-ketoacyl reductase (KAR), wrinkledI1 (WRI1), glycerol-3-phosphate acyltransferase (GPAT), lysophosphatidic acid acyltransferas (LPAT), diacylglycerol acyltransferase (DGAT), long-chain acyl-CoA synthetase (LACS), phosphatidic acid phosphatase (PAP), enoyl-acp reductase (ENR), and fatty acid desaturase (FADs) enzymes are the key regulators of plant lipids [34,35,36,37,38]. To date, the genomes of *M. integrifolia* [39] and *M. tetraphylla* [1] have been sequenced. However, the research progress has been very slow in *M. ternifolia*. The identification of genes involved in lipid biosynthesis and characterization of their expression patterns are two critical prerequisites to reveal the genetic mechanism of lipid biosynthesis in the *M. ternifolia* fruit kernel. Therefore, we designed this study regarding developing the fruit kernels of *M. ternifolia* with the following aims: (1) examine the diversity of lipids content, (2) identify key genes involved in the regulation of lipids, and (3) characterize their expression patterns. This study provides comprehensive insights into lipid composition and associated transcripts in developing fruit kernels. Our data resources are an important foundation to clarify the genetic mechanism of storage lipid accumulation in *M. ternifolia.*

## 2. Results

### 2.1. RNA Sequencing, De Novo Assembly, and Annotations

To obtain the genome-wide transcriptional changes in the fruit kernel of *M. ternifolia*, kernels from three different fruit developmental stages (young (S1-1,1-2,1-3), medium-aged (S2-1,2-2,2-3), and mature (S3-1,3-2,3-3) fruit) were selected to perform RNA sequencing. The mean raw reads in S1 were 54,363,432, whereas an average of 48,083,672 and 52,648,740 raw reads were obtained in the S2 and S3 stages, respectively (Table 1). The filtering of low-quality reads produced an average of 52,621,607, 45,606,890, and 50,793,072 clean reads in the libraries of S1, S2, and S3, respectively. A total of 7.9 Gb of clean data was obtained from the S1 stage, while 6.8 and 7.6 Gb were acquired from stages S2 and S3, respectively. The Q30% was 93% and the GC content percentage was almost 45 in our sequencing. This result suggested that we had produced high-quality data that was suitable for performing transcriptome downstream analysis. Trinity software was used to assemble the 307,336,019 total bases. The total number of unigenes assembled was 279,972. The average length was 1098 nt, with N50 and N90 length values of 1633 and 432 nt, respectively (Supplementary Materials Table S1).

**Figure 1:**
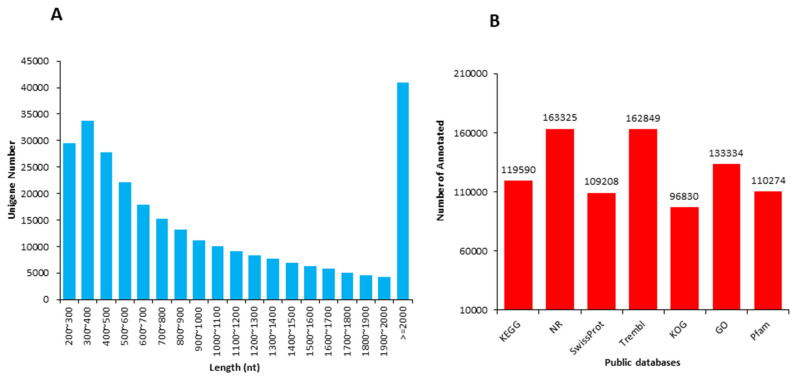
Length distribution and functional annotations of unigenes in *M. ternifolia* fruit kernels: (**A**) Overall length distribution of sequences and (**B**) functional annotations for unigenes in public databases.

**Figure 2:**
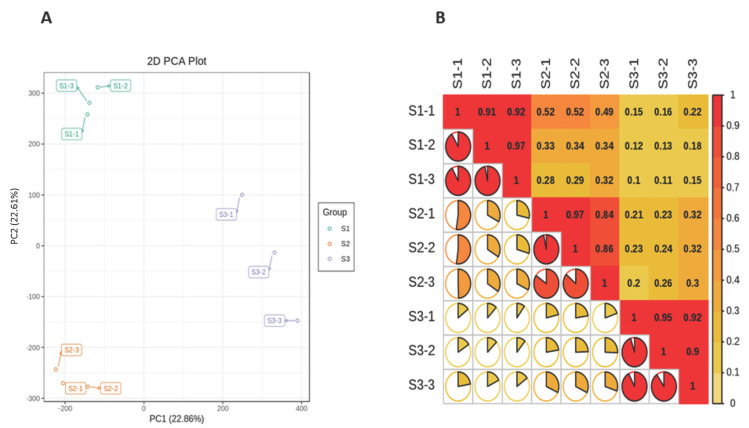
Principal component analysis and correlation of *M. ternifolia* fruit kernels: (**A**) principal component analysis and (**B**) correlation analysis between the samples.

The length distribution range of the unigenes was 200 to 2000 nt (Figure 1A). Due to the lack of a whole reference genome of *M. ternifolia*, all unigenes were blasted by using Nr, Swiss-Prot, KEGG, KOG, GO, and Trembl public databases to obtain annotations (Figure 1B). The majority of unigenes showed functional annotation in the Nr (163,325) and Trembl (162,849) databases. There were 119,590, 109,208, 96,830, 133,334, and 110,274 unigenes that were annotated in KEGG, SwissProt, KOG, GO, and Pfam, respectively. The abundance of each unigene was measured with fragments per kilobase per million reads (FPKM) values. The experiment and sampling suitability was determined with principal component analysis (PCA) and correlation matrixes. The PCA showed that the S1, S2, and S3 fruit kernel stages had significant variations with three distinct clusters (Figure 2A). However, a weak variation among replicates of each stage was observed. The difference was equally covered by PC1 and PC2, which explained 22.9 and 22.6% of the total variation, respectively. Moreover, a strong correlation (R^2^ > 0.90) was observed between the replicates of each stage (Figure 2B). In contrast, a weaker correlation was determined between different fruit kernel stages. The correlation value between S1 and S2 was around 0.50, whereas S1 and S3 showed a much weaker correlation (R^2^ = 0.15). The lower degree of correlation between the three stages indicated strong transcriptional changes.

### 2.2. Transcriptome Changes in Different Fruit Kernel Stages

**Figure 3:**
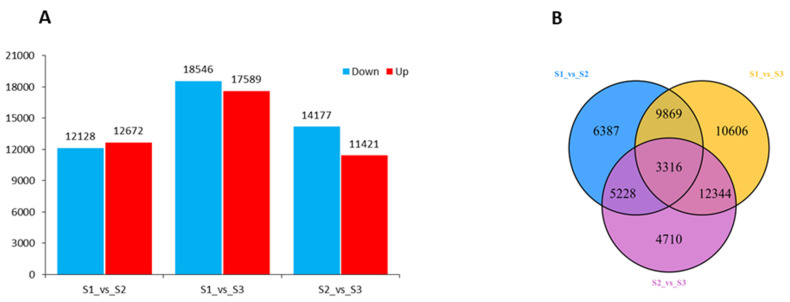
Distribution of total DEGs in different developing stages of *M. ternifolia* fruit kernels: (**A**) division of up- and downregulated DEGs and (**B**) division of overlapped and unique DEGs.

To explore the gene expression changes in developing fruit kernels, we compared the young (S1), medium-aged (S2), and mature (S3) fruit kernel stages. Differentially expressed genes (DEGs) were obtained for two compared samples using *p* ≤ 0.05 and fold changes (ratio ≥ 1 or ratio ≤ −1) as thresholds (Figure 3A). The comparison between S1 and S2 resulted in 12,128 downregulated and 12,672 upregulated DEGs. A total of 36,135 DEGs were determined in the comparison of S1 and S3. From these, 18,546 genes were downregulated and 17,589 genes were upregulated. When we compared S2 and S3, 14,177 genes were downregulated and 11,421 genes were upregulated, with a total of 25,598 DEGs. The distribution of DEGs revealed that a major portion of the DEGs was overlapped but less stage-specific DEGs were identified among the different fruit kernel stages (Figure 3B). For example, the number of overlapped DEGs in S1 vs. S2 and S1 vs. S3 was 13,185. In addition, 3316 DEGs were overlapped among the S1, S2, and S3 fruit kernel stages. Globally, these results indicate major gene expression dynamics for fruit kernel development in *M. ternifolia*.

### 2.3. Functional Enrichment Analysis of DEGs

**Figure 4:**
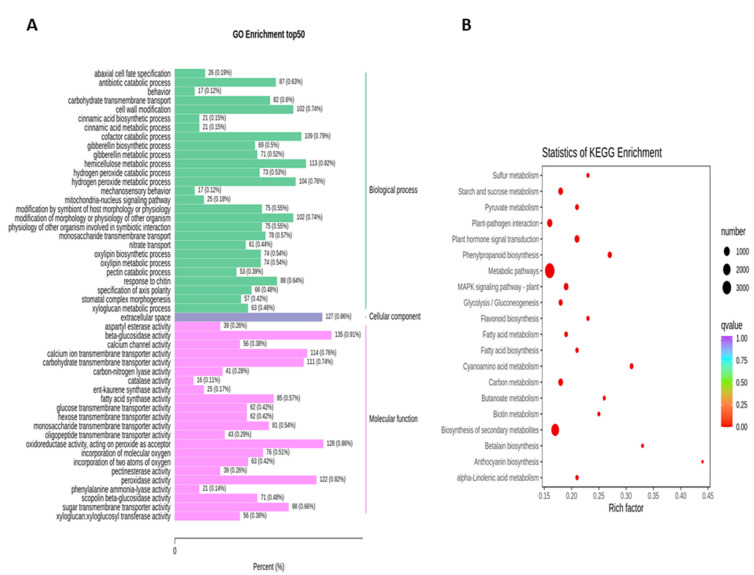
Functional and pathway enrichment analysis for DEGs in the young and medium-aged (S1 vs. S2) stages of *M. ternifolia* fruit kernels: (**A**) functional enrichment and (**B**) pathway enrichment.

**Figure 5:**
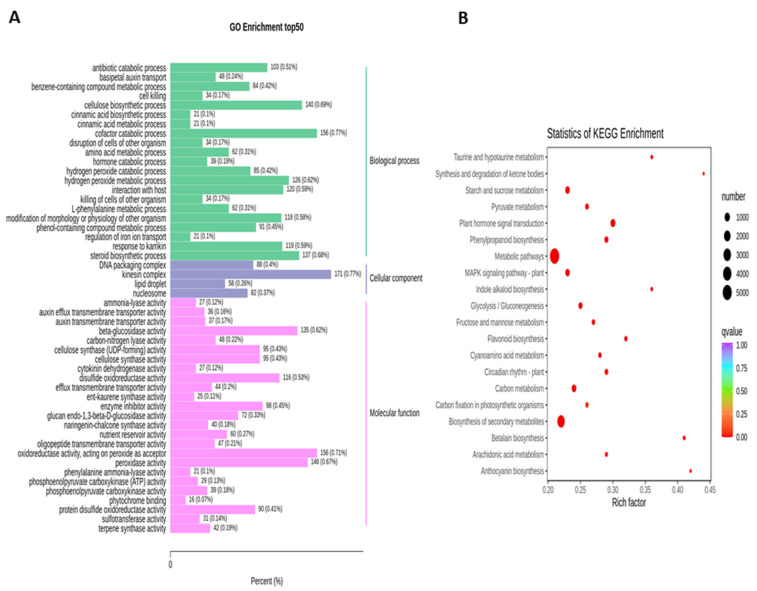
Functional and pathway enrichment analysis for DEGs in the young and mature (S1 vs. S3) stages of *M. ternifolia* fruit kernels: (**A**) functional enrichment and (**B**) pathway enrichment.

**Figure 6:**
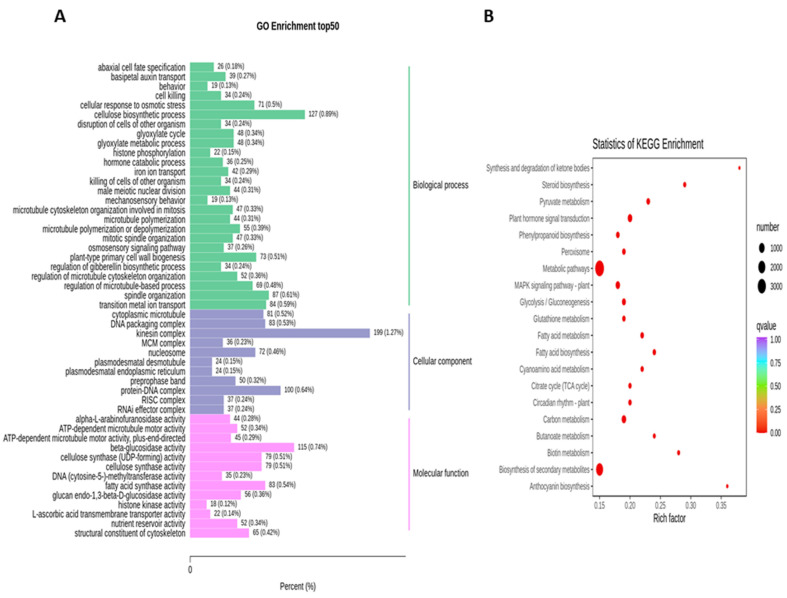
Functional and pathway enrichment analysis for DEGs in the medium-aged and mature (S2 vs. S3) stages of *M. ternifolia* fruit kernels: (**A**) functional enrichment and (**B**) pathway enrichment.

To obtain the complete functional information about transcripts that regulate fruit kernel development, GO and KEGG enrichment analyses were performed by using *p* ≤ 0.05 as the criterion for significant enrichment. In the GO analysis, the majority of DEGs in the S1 and S2 stages were involved in the molecular function of beta-glucosidase and oxidoreductase activity (Figure 4A). Meanwhile, in pathway enrichment, metabolic and biosynthesis of secondary metabolites had more significance and total genes. A total of 1595 DEGs were enriched in each of these pathways (Figure 4B). High numbers of oxidoreductase- and peroxidase-activity-associated genes were observed in the DEGs from the comparison of S1 vs. S3 (Figure 5A). DEGs with annotations regarding metabolism and biosynthesis of secondary metabolites exhibited predominant pathway enrichment (Figure 5B). Functional enrichment analysis of the S2 vs. S3 stages revealed that the majority of DEGs performed the biological function of cellulose biosynthesis, the molecular function of beta-glucosidase, and the cellular function of the kinesin complex (Figure 6A). The majority of DEGs in the S2 vs. S3 stages had the same pathway enrichment as the S1 vs. S2 and S1 vs. S3 stages (Figure 6B). These findings suggested that genes involved in the functional activity of beta-glucosidase and oxidoreductase made significant contributions to the fruit kernel development in *M. ternifolia*.

### 2.4. Overview of the Lipid Composition at Different Fruit Kernel Developmental Stages

**Figure 7:**
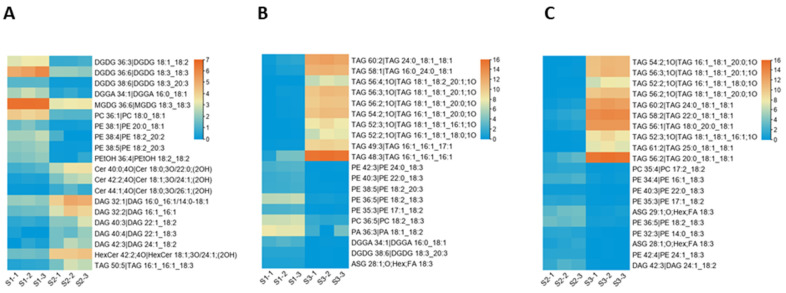
Heatmaps of the lipid content that had the highest differential accumulation among the different stages of the *M. ternifolia* fruit kernels: (**A**) in the comparison of the young (S1) and medium-aged (S2) stages, (**B**) in the comparison of the young (S1) and mature (S3) stages, and (**C**) in the comparison of the medium-aged (S1) and mature (S3) stages.

The kernel of *M. ternifolia* has more than 60% oil content. To understand the lipid diversity and content in the fruit kernel, we analyzed the lipid profiles at different fruit kernel growth stages. In total, 408 different types of lipids were detected in our study (Supplementary Materials Table S2). Most of these lipids were categorized as glycerolipids, sterol lipids, sphingolipids, glycerophospholipids, and fatty acyls. These categories comprised major classes, including 118 triacylglycerols (TAGs), 31 diacylglycerols (DAGs), 50 ceramides (CERs), 4 sulfoquinovosyl diacylglycerols (SQDGs), 4 sphingomyelins (SMs), 4 stigmasterol hexosides (SGs), 14 acylated sterol glycosides (ASGs), 11 phosphatidylmethanols (PMeOHs), 19 phosphatidylcholine (PCs), 35 phosphatidylethanolamines (Pess), 11 phosphatidylglycerols (PGs), 10 phosphatidylinositols (PIs), 3 phosphatidic acids (PAs), 6 monogalactosyldiacylglycerols (MGDGs), 20 hexosyl ceramides (HexCers), 22 fatty acyls (FAs), 5 diacylglycerol glucuronides (DGGAs), and 12 digalactosyl diacylglycerols (DGDGs). It was observed that the contents of 155 subclasses of lipid were altered in the S2 stage compared with S1. Among all of these subclasses, 58 had high accumulation at S2 and 97 lipids exhibited a lower concentration during the S2 stage compared with S1 (Supplementary Materials Table S3). Moreover, three CERs, namely, 40:0, 42:2, and 44:1 subclasses, showed the highest fold change values (Figure 7A). All these lipids had a high concentration in the S2 stage of the fruit kernel. In addition, DAGs 32:1, 32:2, 40:3, 40:4, 42:3; HexCer 42:2; DGGA 34:1; and TG 50:5 lipid species were upregulated in the S2 stage. The top ten lipid subclasses with the lowest fold change values in the S2 stage were three DGDGs (36:3, 36:6, 38:6), four PEs (36:4, 38:1, 38:4, 38:5), MGDG 36:6, PC 36:1, and TAG 50:5 (Figure 7A).

The comparative analysis between the S1 and S3 stages revealed 261 lipid subclasses with differential concentrations (Supplementary Materials Table S4). From these, 187 had a high accumulation in S3 compared to S1 and only 74 subclasses of lipid showed a lower content in S3. Quantification showed that TAGs, including 49:3, 52:2, 52:3, 54:2, 56:2, 56:3, 56:4, 58:1, 60:2, and 62:2, had their highest levels in the S3 stage as compared with S1 (Figure 7B). Meanwhile, the top ten lipids with the lowest accumulation content in the S3 stage belonged to different subclasses (Figure 7B). For example, PEs 35:3, 36:5, 38:5, 40:3, 42:3; ASG 28:1; DGDG 38:6; DGGA 34:1; PA 36:3; and PC 36:5 exhibited higher contents during the S1 stage than S3. In total, 404 lipid subclasses had differential content accumulation in S3 compared to S2 (Supplementary Materials Table S5). A total of 203 lipids were obtained with a higher level, whereas 201 had a lower level in the S3 stage. Further results suggested that TAGs, such as 52:2, 52:3, 54:2, 56:1, 56:2;1O, 56:2, 56:3, 58:2, 60:2, and 61:2 had a significantly increased level in S3 than S2 (Figure 7C). In contrast, PEs 32:3, 34:4, 35:3, 36:5, 40:3, 42:4; ASGs 28:1, 29:1; DG 42:3; and PC 35:4 were highly reduced in the S3 stage (Figure 7C). It was noticed that there were few stage-specific lipids but there were more overlapped or common lipid profiles among the kernel developmental stages (Supplementary Materials Figure S2). Overall, the results of the lipid profiling revealed that during fruit kernel growth, triacylglycerols and diacylglycerol related lipids accumulation were increased. In contrast, phosphatidylethanolamine-associated lipids showed decreased accumulation.

### 2.5. Lipid-Related Gene Expression Changes during Fruit Kernel Development

To find the key genes associated with fruit kernel development, further mining of metabolic genes was achieved through the selection of DEGs having a fold change value ≥ 5 or ratio ≤ −5 as thresholds. The Venn diagram revealed 282 stage-specific highly expressed DEGs between S1 and S2 (Supplementary Materials Figure S2). The S1 and S3 stages comparison had 246 stage-specific DEGs. It was observed that S2 vs. S3 had 412 stage-specific DEGs with a high fold change ratio. Moreover, 400 overlapped DEGs among all stages were considered as a regulator of the metabolic process during fruit kernel growth. The expression trends of annotated genes associated with lipid biosynthesis are shown in Table 2. Significant low expression levels of four genes related to 3-ketoacyl-acyl carrier protein reductase (*unigene 73227*, *unigene 211387*, *unigene 73228*, *unigene 26385*) were observed during the S3 fruit kernel developmental stage compared to the S1 stage. The expression levels of five beta-glucosidase genes, namely, *unigene 170150*, *unigene 81638*, *unigene 81642*, *unigene 81652*, and *unigene 91684*, were highest during the S2 stage. Similar results were obtained for *unigene 150581*, *unigene 150580*, and *unigene 140441*, which encode the enoyl-acyl-carrier-protein reductase (NADH) enzyme. In addition, the diacylglycerol-kinase-2-linked *unigene 147364* had an unpredictable expression during the fruit kernel development stage. A significantly altered expression was also determined for pyruvate-kinase-2-annotated *unigene 63669*, *unigene 63675*, and *unigene 86831* during fruit kernel growth.

Triacylglycerols are the key resources of energy in the seeds. Two unigenes encoding glycerol-3-phosphate acyltransferase and denoted as *unigene 152617* and *unigene 128368* were found with low expressions during the late stage of fruit kernel growth in our study. Moreover, a significant change in the expression trends of some genes related to GDSL esterase lipase was obtained during the fruit kernel developmental stages. For example, *unigene 240247*, *unigene 82774*, *unigene 183098*, *unigene 38911*, *unigene 113245*, and *unigene 238268* showed increased expression levels during the S1 stage compared with S2 and S3. In contrast, *unigene 213504*, *unigene 62756*, *unigene 6967*, and *unigene 103298* had higher levels of expression in the S2 stage than S1 and S3. Globally, the expression of linoleate 13S-lipoxygenase 2-1 gene family members, such as *unigene 9629*, *unigene 66716*, *unigene 54520*, *unigene 59928*, *unigene 213589*, *unigene 37584*, *unigene 34267*, *unigene 13471*, *unigene 5404*, and *unigene 139752*, were significantly downregulated during the late stages of fruit kernel growth. Various genes involved in phospholipids regulation were determined with altered expression levels during the fruit kernel growth. Among these, *unigene 190439*, *unigene* 227869, and *unigene 171746* had higher expression values in the S2 stage compared with S1 and S3, whereas *unigene 158347* and *unigene 122230* showed upregulated expression during the S1 (early fruit kernel growth) stage. Overall, this suggests that the stage-specific regulation of genes related to lipid metabolism and catabolism caused the diversity of lipid compositions and concentrations in *M. ternifolia* nuts.

### 2.6. Validation of Key Genes Involved in Lipid Regulation Using qRT-PCR Analysis

Based on the transcript downstream data analysis, ten genes putatively involved in the lipid regulation were selected to perform a real-time qRT-PCR analysis in order to confirm the accuracy of the RNA-seq. The selected genes showed differential expressions at the different developmental stages of the fruit kernel in *M. ternifolia* (Supplementary Materials Figure S3). It was observed that the expression trends of all genes were consistent with RNA-seq, thus confirming the reliability of RNA-seq results in our study.

## 3. Discussion

Out of four cultivated species, *M. integrifolia* and *M. tetraphylla* are widely grown in commercial orchards [40]. There are many constraints that limit the productivity of *M. ternifolia.* The major challenge is a high level of cyanogenic glycosides present in the kernel, which are toxic and unsuitable for human consumption without steeping and cooking [4,41]. A detailed understanding of the genetic mechanisms underlying lipid composition in the kernel of *M. ternifolia* will be useful to promote its commercial production level. Through comprehensive lipidomics and transcriptomic data analyses, this study improved our understanding of the molecular factors that influence oil accumulation in *M. ternifolia* kernels.

### 3.1. Divergence of Lipid Composition in the Kernels of M. ternifolia

A lipid is a form of stored carbon in plant seeds, yields more energy than carbohydrates, and is an integral part of plant organs [29]. The lipid composition was significantly changed in the developing fruit kernels of *M. ternifolia.* In our study, a total of 407 different types of lipid species were determined at different fruit kernel growth stages. The majority of these lipids belonged to the category of glycerolipids including triacylglycerols (TAGs). TAGs are the primary components of Macadamia oil, accounting for more than 95% of total lipids. These influence the nutritional characteristics of nut oil through physical and biochemical changes [42]. TAGs are entirely acylated derivatives of glycerol and the most abundant neutral lipid bodies present in seeds [43], senescence leaves, and pollen grains [44,45]. In the mature fruit kernels of *M. ternifolia*, the most accumulated species of TAGs were long-chain unsaturated fatty acids, including TAG 52:2;1O, TAG 54:2;1O, TAG 56:3;1O, and TAG 56:2;1O. An increase in unsaturated TAGs was previously observed in the later stages of seed development in *Paeonia ostii* [46], *Glycine* max [47], and *Brassica napus* [48]. The stored level of TAGs has a functional linkage to seed germination [49] and stress management [50]. Moreover, the nuts of Macadamia species are usually consumed in roasted form, which can modify the lipid profiles due to oxidation [51]. Excessive oxidation in unsaturated TAGs produces free fatty acids due to esterases and lipases through the activity of hydrolytic enzymes [52]. Free fatty acids are more vulnerable to oxidation during nut roasting, which causes adverse chemical changes through rancidity and the deterioration process, which ultimately leads to rejection by consumers due to a lower quality of nuts [53]. In a recent study, it was observed that roasting had a non-significant impact on the TAGs composition and it ultimately improved the flavor, aroma, color, texture, and appearance of macadamia nuts [42]. Among other detected glycerolipids in the developing fruit kernels of *M. ternifolia*, different species of diacylglycerols (DAGs) have a high abundance. In particular, DAGs 40:4 and 42:3 species had a high level of accumulation. DAGs are precursors of glycolipids, are swiftly phosphorylated to produce phosphatidic acid (PA) by diacylglycerol kinase enzyme, and act as a potential secondary messenger in plants [54]. The high contents of TAGs and DAGs in the fruit kernel of *M. ternifolia* may be useful to improve the oil quality through the biosynthesis of other essential fatty acids and lipids.

Phosphatidylethanolamine (PE) is a polar lipid belonging to the phospholipid category, is dominant in the plasma membrane, and plays an important role in cold stress tolerance [55]. In our study, the quantitative level of many PE species was dramatically reduced at the mature fruit kernel stage. Specifically, PE 35:3, PE 36:5, PE 40:3, PE 42:4, and PA 36:3 had higher levels of accumulation in the young fruit kernel of *M. ternifolia*. Previous research showed that Pes, together with phosphatidylcholine species, alleviates chilling injury to protect cell membrane damage in cold conditions [56]. PA is a type of glycerophospholipid that mediates the biosynthesis of phospholipids and glycolipids in plants. Moreover, it is involved in secondary signal transduction during abiotic stress, including salinity [57], drought [58], and cold [59]. Our analysis further reported a differential level of ceramides (CERs) in the medium fruit kernel stage, in particular, high levels of Cer 44:1 and Cer 42:2 contents were detected. CERs are sphingolipids that are essential components of the membrane in plants. Functional studies showed that CERs are involved in responses to biotic and abiotic stimuli. However, little is known about their signaling mechanism during the programmed cell death of certain bacteria and fungi pathogens [60]. PEs, PAs, and CERs have a critical role in the defense against various stressful conditions. A similar function in developing the kernel of *M. ternifolia* might be linked with a higher level of lipid stores in the nut. The data of lipidomic analysis identified the presence of some species of acylated sterol glycosides, phosphatidylmethanol, diacylglyceryl glucuronide, and digalactosyldiacylglycerol in the kernel of *M. ternifolia*. However, their non-differential trend suggested a minor role for the oil quality index. In contrast, a higher abundance of oil species, such as TAGS, DAGs, PEs, and PCs, in the mature nut is of substantial importance to the improve oil quality in *M. ternifolia* nut.

### 3.2. Lipid Biosynthesis in Fruit Kernels of M. ternifolia

Lipid biosynthesis in nuts is predominantly altered by the activity of genes that are involved in the de novo biosynthesis of fatty acids, synthesis of triacylglycerol, and formation of oil bodies [35]. The genetic mechanism of fatty acid and lipid synthesis leading to TAG is well known in Arabidopsis [61]. The substrate for all synthesized fatty acid pools is acetyl-CoA. The enzyme pyruvate kinase (PK) in the plastids generates pyruvate, which is catalyzed by the plastidial pyruvate dehydrogenase complex to form acetyl-CoA for the de novo production of fatty acids [62]. The altered expression of *PK2* genes in young fruit kernels of *M. ternifolia* suggests their vital role in the generation of diverse fatty acids. Functional genomics experiments on *PK2* genes in rice stated their importance in fatty acid and starch metabolism [63]. Among the fatty acid synthase system, genes associated with 3-ketoacyl-acyl carrier protein reductase (KAR) or synthase and enoyl-acyl-carrier-protein reductase (NADH) (ENR) regulate the synthesis of long-chain unsaturated fatty acids in plants seeds [64]. In the formation of long-chain fatty acids, the KAR enzyme reduces 1,3-ketobutyryl-ACP to 3-hydroxybutyryl-ACP, which extends a hydrocarbon chain of the acyl group up to a 16- or 18-carbon chain through a series of cycle reactions [65]. Similarly, ENR is predicted to be involved in the last step of fatty acid elongation in plants, bacteria, and mammals [66]. The altered expression of genes involved in the functional activity of KAR and ENR enzymes probably contributes to the development of unsaturated fatty acids in *M. ternifolia*. In this way, it might be interlinked with the unique biochemical and physical properties of nut oil. However, further research evidence is needed to support this statement. In our lipidomic data analysis, TAGs, DAGs, PEs, and PAs are the most synthesized forms of lipids present in the fruit kernel of *M. ternifolia*. The de novo lipid biosynthesis of *M. ternifolia* most probably starts with the catalytic activity of glycerol-3-phosphate acyltransferase (GPAT) enzyme. The GPAT initiates the preliminary reaction of TAG and phospholipids biosynthesis in the endoplasmic reticulum. It further converts glyerol-3-phosphate into lysophosphatidic acid, which produces phosphatidic acid. The dephosphorylation of phosphatidic acid forms DAG and finally TAG [67]. Furthermore, the oleoyl CoA transferred from plastids is used as a substrate to produce membrane lipids (PCs, PEs, and PIs) or the storage lipid TAG in *M. ternifolia.* However, further metabolic studies will be essential to prove these findings.

Several studies have reported the role of phosphoglycerolipids in lipid signaling [68,69]. The key genes involved in lipid signal transduction include diacylglycerol kinase (*DGK*), phosphatidylinositol (*PI*), nonspecific phospholipase C (*NPC*), phospholipase C (*PLC)*, phospholipase D (*PLD*), and *PA* [70]. Interestingly, we observed substantially altered transcription levels of *GPAT*, *DGK*, *PI*, and *NPC* in developing fruit kernel *M. ternifolia*. This indicates their key roles not only in higher TAGs, DAGs, PE, and PA accumulation but also in lipid signaling during the growth of *M. ternifolia* kernel. It was reported that enzyme GDSL-type esterase/lipase can hydrolyze thioesters, aryl esters, phospholipids, and amino acids. In this way, they altered the mechanism of secondary metabolism [71]. A significant change in expression trends of genes related to the GDSL esterase lipase enzyme probably performed a similar function in developing the *M. ternifolia* fruit kernel. Furthermore, our research also identified several genes associated with the linoleate 9S-lipoxygenase (LOX) enzyme, particularly *LOX6*, which had a significantly modified expression in the *M. ternifolia* fruit kernel. This enzyme utilizes linoleic acid/linolenic acid as a substrate to produce unsaturated fatty acids, which improves the oil content and quality for human health [72]. In addition, *LOX* genes play a critical role in the enzymatic oxidation of polyunsaturated fatty acids to prevent germination during seed aging [73]. The β-glucosidases (BGlu) enzymes have numerous roles in plants, such as defense, cell wall lignification, and phytohormone activation. These enzymes are also responsible for the release of scented volatile compounds in plants [74,75]. The increased expression of *BGlu* genes in the developing fruit kernel of *M. ternifolia* suggests an association with the diversity of functions that directly or indirectly affect oil quality.

Concisely, our comparative lipidomic and transcriptomic data analysis identified key regulatory genes associated with oil content and quality in *M. ternifolia* kernels. Due to the wide public acceptance of Macadamia nuts as a portion of quality food, these results are vital for promoting the nutritional and commercial value of *M. ternifolia* kernels. Our results provide a baseline for further transgenic and genetic engineering research with the aim to identify oil candidate genes and their regulators. Hence, further works are needed to boost the application of *M. ternifolia* nuts in the food, pharmaceutical, and cosmetic industries.

## 4. Materials and Methods

### 4.1. Plant Materials, RNA Extraction, and Sequencing

The plant material used in this study was collected from *M. ternifolia* plants cultivated in the Yunnan province of China. The developing fruit kernels comprised of young (S1), medium-aged (S2), and mature (S3) fruiting stages were picked in three biological repeats. The harvested samples were immediately placed in liquid nitrogen and stored at −80 °C before further processing. Total RNA was isolated from all nine samples with a Trizol reagent (Invitrogen, San Diego, CA, USA) following the manufacturer’s standard protocol. RNase-free DNase I (TaKaRa, Kyoto, Japan) was used to purify the total RNA. The RNA quality and quantity were determined by 1% agarose gel and Nanodrop spectrophotometer (Nanodrop Technologies, Wilmington, DE, USA), respectively. Sequencing libraries were prepared with the Illumina TruSeq Standard mRNA library preparation kit following the manufacturer’s standard protocol. After the quality tests, the Illumina HiSeq Ten X platform (Illumina Inc., San Diego, CA, USA) was used according to the recommended protocol to perform de novo pair-end RNA sequencing.

### 4.2. Transcript Assembly, Annotation, and Analysis of DEGs

By removing the adaptors, ambiguous bases, and low-quality reads, the original sequencing data in the form of raw reads were filtered to obtain high-quality clean reads. Because of the non-availability of the reference genome of *M. ternifolia*, high-quality clean reads were assembled into unigenes for subsequent analysis with Trinity 2.6.6 software [76]. The BLAST software was used to compare the unigenes sequence with the Kyoto Encyclopedia of Genes and Genomes database (KEGG), NCBI non-redundant protein sequences (NR), Gene Ontology (GO), euKaryotic Ortholog Groups (KOG), Protein family (Pfam), Trembl, and Swiss-Prot databases to retrieve the unigenes’ functional annotations [77]. The expression levels of the mapped transcript were determined in the form of fragments per kilobase of transcript per million fragments mapped (FPKM) values. The R package DESeq2 version 1.22.2 [78] was used to obtain the total number of differential genes and the number of upregulated and downregulated genes among two different samples. The significant criteria for DEGs included log2 fold change ≥1 and ≤−1 and a false discovery rate < 0.05. The KEGG for linking genomes to life and the environment platform was used to perform the pathway enrichment analysis [79].

### 4.3. Real-Time qRT-PCR Analysis

To perform the qRT-PCR analysis for ten selected genes, the cDNAs were produced with iScript cDNA Synthesis Kit (BIO-RAD). Primer Premier 5.0 was used to design the gene-specific reverse and forward primers (Table S6). The reaction mixture in three technical repeats was prepared with iTaq Universal SYBR Green Supermix 50 mL (BIO-RAD) kit. The quantitative PCR analysis was conducted in triplicate biological replicates on a BIO-RAD iCycler iQ5 real-time PCR system. The *Actin2* gene was used as an internal control. The running and data analysis protocol of the qRT-PCR was followed as detailed in a previous report [80].

### 4.4. Lipid Extraction and Analysis Using UPLC-MS/MS

Different fruit kernels consisting of young (S1), medium-aged (S2), and mature (S3) fruiting stages were utilized to extract lipids. In brief, a 15 mg sample was taken to make a fine powder. First, 2 mL of precooled (−20 °C) methanol was added, followed by 4 mL dichloromethane, and the mixture was vortexed for 1 h. After 10 min of incubation at 4 °C and sonication for 10 min in an ultra-sonication bath, 1.6 mL double-distilled water was added. Samples were then centrifuged for 5 min. Two phases were produced after the centrifugation. The upper clean and residual-free lipophilic phase was carefully removed. The lipophilic phase was then dried under a vacuum. Later, 1 mL isopropyl alcohol was added in the lipophilic phase and used for further lipidomic analysis. The lipid profiling was executed on a 6600 plus AccurateMass Q-TOF (AB Sciex TripleTOF^®^ 6600, Framingham, MA, USA) mass spectrometer system. The process of liquid chromatography was completed using UPLC with an autosampler LC-30A (Shimadzu Corporation) column (100 × 2.1 mm, 2.6 m). The lipid measurement protocol was followed as in the description of Wan et al. [81]. The MSDIAL ver. 4.0, PeakView2.1, and MultiQuant 3.0 were used to perform the quantitative and qualitative data analyses of the lipids [82].

## 5. Conclusions

Our results revealed lipid and transcript profiles at various developing fruit kernel stages of *M. ternifolia* nuts. Moreover, the key lipids and their putative candidate genes were identified. In particular, *GPAT*, *DGK*, *PI*, *NPC*, *PK*, *KAR*, and *LOX* enzymes encoding genes were most probably rate-limiting for the biosynthesis of glycerolipids and phospholipid in *M. ternifolia* nuts. These results help to understand lipid diversity and provide first insights into the genetic mechanism of lipid accumulation. Gene functional characterization and further transcript data from different genotypes are mandatory to explore the regulatory network of lipid biosynthesis in *M. ternifolia* nuts.

## Figures and Tables

**Table 1:** Overview of the transcriptome sequencing dataset and quality check.

Sample	Raw Reads	Clean Reads	Clean Base (Gb)	Mapped Reads (%)	Q20 (%)	Q30 (%)	GC (%)
S1-1	55,604,268	53,433,146	8.0	85	97.9	93.8	44.8
S1-2	56,740,048	54,978,858	8.3	83	97.5	92.8	45.4
S1-3	50,745,980	49,452,818	7.4	83	97.9	93.7	45.2
S2-1	49,242,862	45,820,088	6.8	82	97.8	93.5	44.1
S2-2	46,406,958	44,626,700	6.7	82	97.8	93.5	44.3
S2-3	48,601,196	46,373,882	7.0	81	97.9	93.7	44.1
S3-1	55,107,466	53,593,048	8.0	79	97.8	93.6	45.7
S3-2	46,080,428	44,529,822	6.7	78	97.8	93.6	45.2
S3-3	56,758,326	54,256,348	8.1	76	98.0	94.2	44.0

**Table 2:** Expression profile of genes that encoded enzymes involved in lipid biosynthesis in the *M. ternifolia* kernels.

ID	S1	S2	S3	Annotations
Unigene 73227	61.9	1.8	0.0	3-ketoacyl-acyl carrier protein reductase
Unigene 211387	11.5	0.2	0.0	3-ketoacyl-acyl carrier protein reductase
Unigene 73228	10.6	0.0	0.0	3-ketoacyl-acyl carrier protein reductase
Unigene 163200	0.0	1.2	0.0	3-ketoacyl-acyl carrier protein reductase
Unigene 26385	41.6	0.0	0.0	3-ketoacyl-acyl carrier protein reductase
Unigene 170150	108.4	640.6	3.6	Beta-glucosidase 11
Unigene 41085	27.4	0.2	0.3	Beta-glucosidase 11
Unigene 81638	0.3	18.4	0.0	Beta-glucosidase 12
Unigene 81652	1.3	22.1	0.0	Beta-glucosidase 12
Unigene 91684	0.0	0.8	0.0	Beta-glucosidase 44
Unigene 35002	2.6	0.1	0.0	Beta-glucosidase 41
Unigene 101847	0.0	0.0	1.7	Choline/ethanolaminephosphotransferase 1
Unigene 81642	56.3	479.2	0.1	Cyanogenic beta-glucosidase
Unigene 147364	0.0	5.8	2.7	Diacylglycerol kinase 2
Unigene 150581	0.0	0.7	0.0	Enoyl-acyl-carrier-protein reductase (NADH)
Unigene 150580	0.0	1.4	0.0	Enoyl-acyl-carrier-protein reductase (NADH)
Unigene 140441	0.0	3.0	0.0	Enoyl-acyl-carrier-protein reductase (NADH)
Unigene 240247	4.6	0.0	0.1	GDSL esterase lipase
Unigene 213504	3.7	10.1	0.0	GDSL esterase lipase
Unigene 82774	7.5	5.7	0.0	GDSL esterase lipase
Unigene 183098	6.6	0.9	0.0	GDSL esterase lipase
Unigene 62756	0.0	3.2	0.1	GDSL esterase lipase
Unigene 38911	1.4	0.0	0.0	GDSL esterase lipase
Unigene 113245	1.4	0.0	0.0	GDSL esterase lipase
Unigene 238268	12.8	0.5	0.0	GDSL esterase lipase
Unigene 69671	18.5	40.2	0.5	GDSL esterase lipase APG
Unigene 103298	5.3	11.0	0.0	GDSL esterase lipase APG
Unigene 152617	1.5	0.0	0.0	Glycerol-3-phosphate acyltransferase
Unigene 128368	1.1	0.0	0.0	Glycerol-3-phosphate acyltransferase
Unigene 163240	1.8	1.0	0.0	Hydroxymethylglutaryl-CoA synthase
Unigene 122230	71.4	2.0	60.9	Inositol-3-phosphate synthase
Unigene 9629	0.7	0.0	0.0	Linoleate 13S-lipoxygenase 2-1
Unigene 66716	9.1	0.1	0.0	Linoleate 13S-lipoxygenase 2-1
Unigene 54520	1.3	0.0	0.0	Linoleate 13S-lipoxygenase 2-1
Unigene 59928	20.7	0.1	0.0	Linoleate 13S-lipoxygenase 2-1
Unigene 213589	5.5	0.0	0.0	Linoleate 13S-lipoxygenase 2-1
Unigene 37584	19.1	0.3	0.0	Linoleate 13S-lipoxygenase 2-1
Unigene 34267	15.0	0.1	0.0	Linoleate 13S-lipoxygenase 2-1
Unigene 13471	2.4	0.1	0.0	Linoleate 9S-lipoxygenase A
Unigene 5404	0.9	0.0	0.0	Linoleate 9S-lipoxygenase A
Unigene 139752	0.5	0.0	0.5	Lipoxygenase 4
Unigene 227869	4.6	11.8	0.1	Phosphatidylinositol 4-kinase alpha 1
Unigene 171746	72.5	95.5	0.6	Phospholipase A2-alpha
Unigene 56285	0.0	0.0	0.8	Phospholipid hydroperoxide
Unigene 63669	24.8	20.0	0.5	Pyruvate kinase 2
Unigene 63675	2.4	2.4	0.0	Pyruvate kinase 2
Unigene 86831	2.8	1.0	0.0	Pyruvate kinase 2
Unigene 158347	4.21	0.00	0.26	Non-specific phospholipase C6; EC
Unigene 190439	5.87	6.86	0.08	Non-specific phospholipase C2; EC

## Data Availability

The raw transcriptome data were submitted to NCBI SRA under the project number PRJNA761018.

## Supplementary Materials

The following are available online at https://www.mdpi.com/article/10.3390/life11121431/s1, Table S1: Statistics of assembly for the different fruit kernel stages of *M. ternifolia*. Table S2: Total diversity of lipids and their content level in the different fruit kernel stages of *M. ternifolia*. Table S3: Total contents of lipids differentially accumulation in the comparison of the S1 vs. S2 stages. Table S4: Total contents of lipids differentially accumulation in the comparison of the S1 vs. S3 stages. Table S5: Total contents of lipids differentially accumulation in the comparison of the S2 vs. S3 stages. Table S6: Primer sequences used in the qRT-PCR experiment. Figure S1: Distribution of the overlapped and unique number of lipid species among the different fruit kernel stages of *M. ternifolia*. Figure S2: Distribution of the overlapped and unique highly expressed metabolic DEGs among the different fruit kernel stages of *M. ternifolia*. Figure S3: qRT-PCR validation of selected differentially expressed genes. The *Actin2* gene was used as the internal control and the transcript levels in S2 and S3 were expressed as compared with S1. Young (S1), medium-aged (S2), and mature (S3) fruiting stages.
