# Supplementary material for: Combined Transcriptome and Lipidomic Analyses of Lipid Biosynthesis in Macadamia ternifolia Nuts
Source: Life (Basel). 2021 Dec 18;11(12):1431. doi: 10.3390/life11121431 (PMC8707767; doi:10.3390/life11121431)
Supplement: Supplementary file 1 [file life-11-01431-s001.zip › life-1470828-supplementary.pdf]

life

Supplementary material of Combined Transcriptome and Lipidomic Analyses of Lipid Biosynthesis in *Macadamia ternifolia* Nut

Table S1. Statistics of assembly for different fruit kernel stages of M. ternifolia.

**Total Number of Unigene**	**279972**
N50 Length	1633
N90 Length	492
Mean Length	1098
Total Assembled Bases	307336019

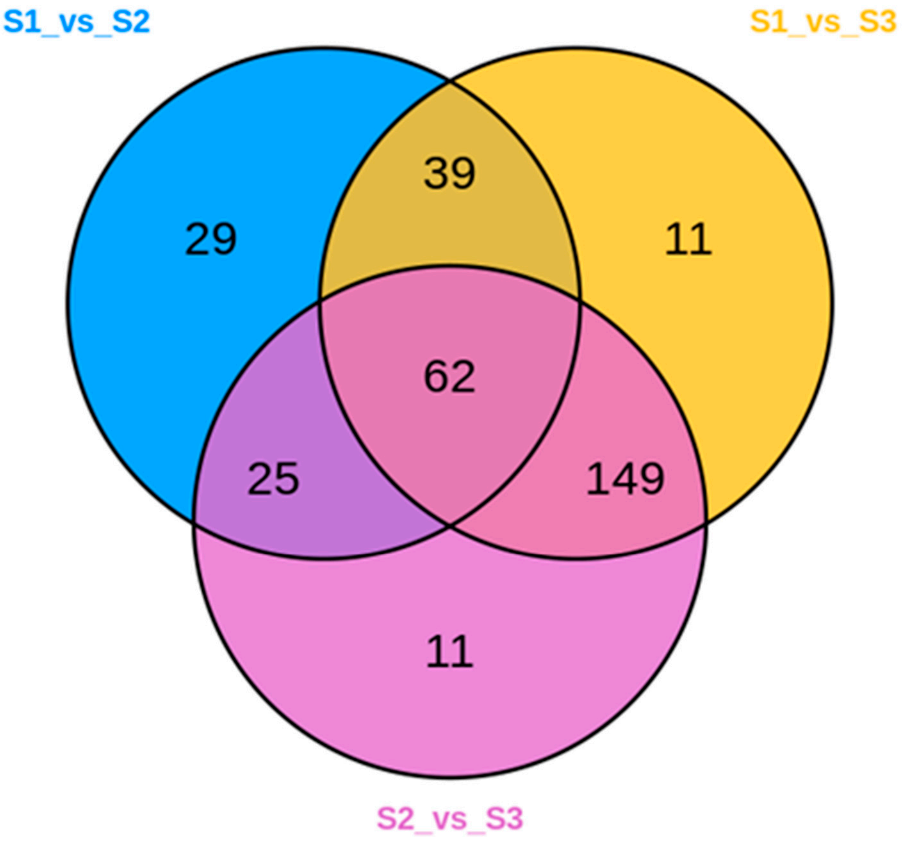

Figure S1. Distribution of the overlapped and unique number of lipid species among the different fruit kernel stages of *M. ternifolia.*

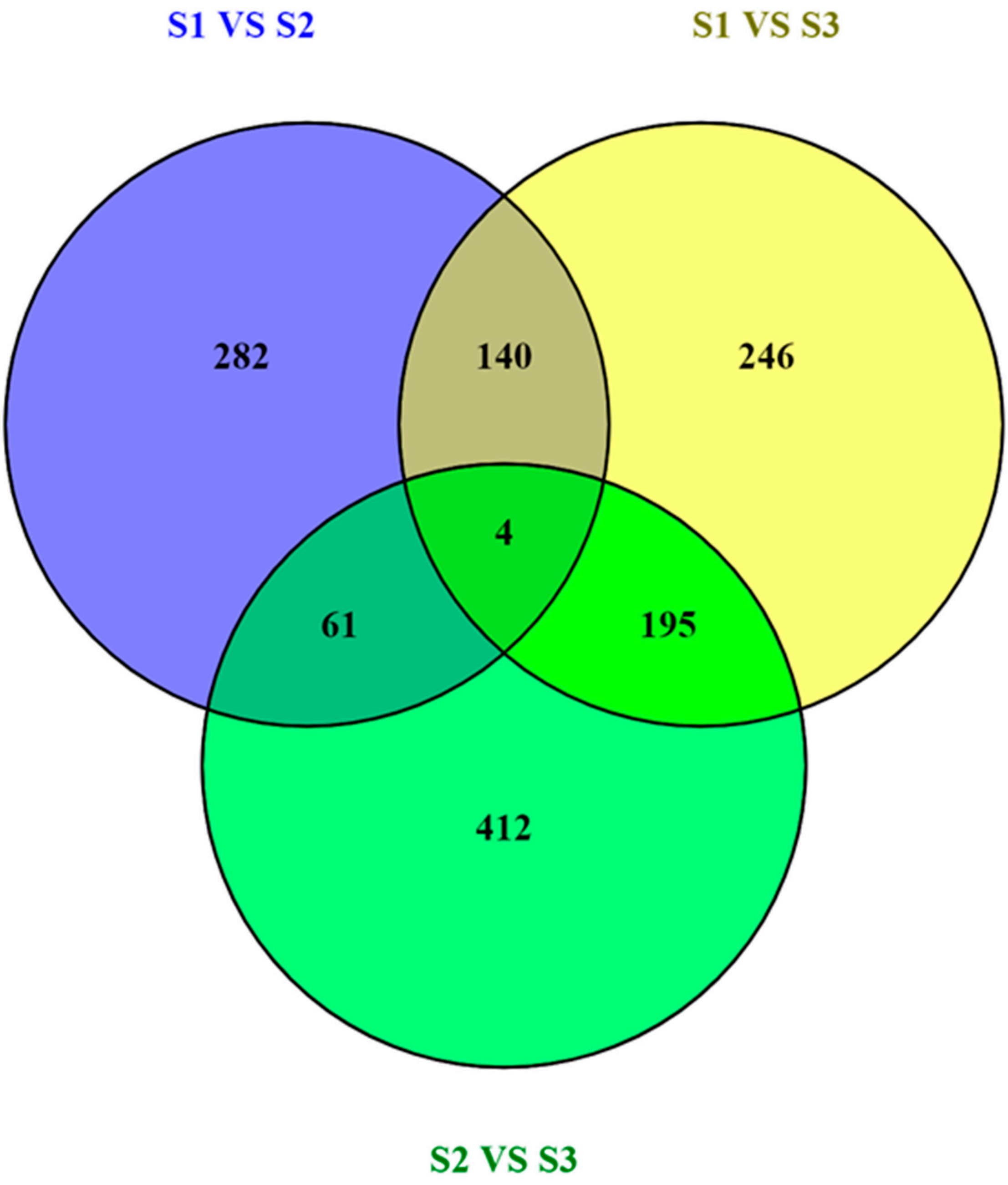

Figure S2. Distribution of the overlapped and unique highly expressed metabolic DEGs among the different fruit kernel stages of *M. ternifolia*.

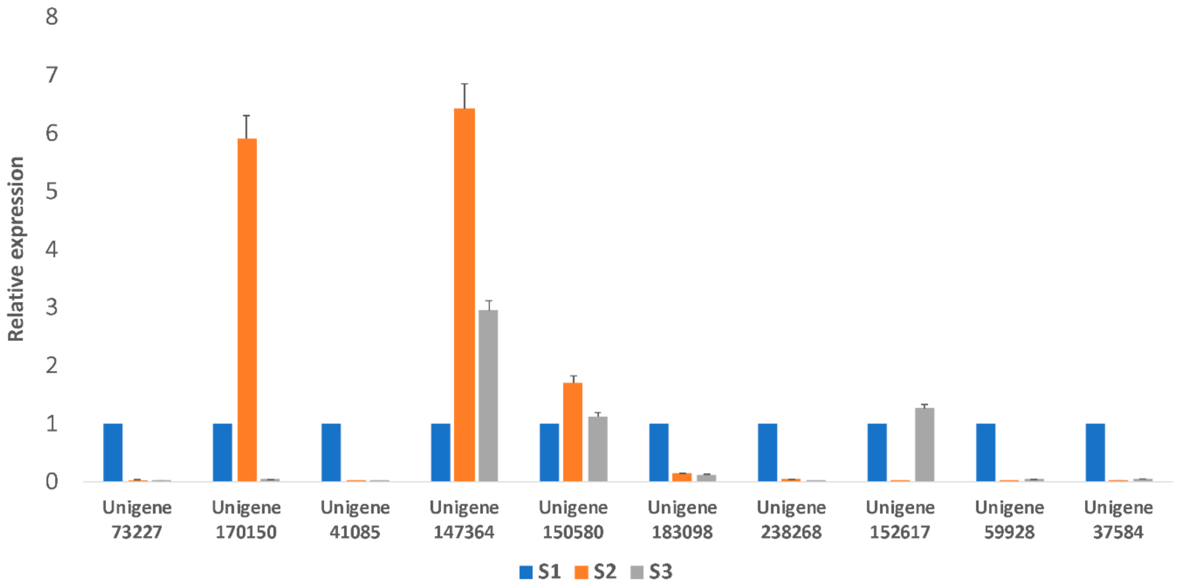

Figure S3. qRT-PCR validation of selected differentially expressed genes. The *Actin2* gene was used as the internal control and the transcript levels in S2 and S3 were expressed as compared with S1. Young (S1), medium-aged (S2), and mature (S3) fruiting stages.
